# Improved homology modeling of the human & rat EP_4_ prostanoid receptors

**DOI:** 10.1186/s12860-019-0212-5

**Published:** 2019-08-27

**Authors:** Melissa C. Holt, Chi S. Ho, M. Inés Morano, Stephen D. Barrett, Adam J. Stein

**Affiliations:** Cayman Chemical Co, 1180 E. Ellsworth Rd, Ann Arbor, MI 48108 USA

**Keywords:** Prostaglandin E_2_, Prostaglandin E_2_ receptor, Prostaglandin E_2_ receptor subtype 4, PGE_2_, EP_4_, Homology model, Structure-based drug design, Bone healing, Heart failure

## Abstract

**Background:**

The EP_4_ prostanoid receptor is one of four GPCRs that mediate the diverse actions of prostaglandin E_2_ (PGE_2_). Novel selective EP_4_ receptor agonists would assist to further elucidate receptor sub-type function and promote development of therapeutics for bone healing, heart failure, and other receptor associated conditions. The rat EP_4_ (rEP_4_) receptor has been used as a surrogate for the human EP_4_ (hEP_4_) receptor in multiple SAR studies. To better understand the validity of this traditional approach, homology models were generated by threading for both receptors using the RaptorX server. These models were fit to an implicit membrane using the PPM server and OPM database with refinement of intra and extracellular loops by Prime (Schrödinger). To understand the interaction between the receptors and known agonists, induced-fit docking experiments were performed using Glide and Prime (Schrödinger), with both endogenous agonists and receptor sub-type selective, small-molecule agonists. The docking scores and observed interactions were compared with radioligand displacement experiments and receptor (rat & human) activation assays monitoring cAMP.

**Results:**

Rank-ordering of in silico compound docking scores aligned well with in vitro activity assay EC_50_ and radioligand binding K_i_. We observed variations between rat and human EP_4_ binding pockets that have implications in future small-molecule receptor-modulator design and SAR, specifically a S103G mutation within the rEP4 receptor. Additionally, these models helped identify key interactions between the EP_4_ receptor and ligands including PGE_2_ and several known sub-type selective agonists while serving as a marked improvement over the previously reported models.

**Conclusions:**

This work has generated a set of novel homology models of the rEP_4_ and hEP_4_ receptors. The homology models provide an improvement upon the previously reported model, largely due to improved solvation. The hEP_4_ docking scores correlates best with the cAMP activation data, where both data sets rank order Rivenprost>CAY10684 > PGE_1_ ≈ PGE_2_ > 11-deoxy-PGE_1_ ≈ 11-dexoy-PGE_2_ > 8-aza-11-deoxy-PGE_1_. This rank-ordering matches closely with the rEP_4_ receptor as well. Species-specific differences were noted for the weak agonists Sulprostone and Misoprostol, which appear to dock more readily within human receptor versus rat receptor.

**Electronic supplementary material:**

The online version of this article (10.1186/s12860-019-0212-5) contains supplementary material, which is available to authorized users.

## Background

Prostaglandin E_2_ (PGE_2_) in humans is an ubiquitous arachidonic acid-cyclooxygenase (COX) cascade product that mediates a multitude of signaling roles through activation of the GPCR superfamily-member E-type prostanoid receptors 1–4 (EP_1–4_) [1]. The roles of functionally related EP receptors have been widely investigated, and the receptors have been considered as therapeutic targets for a variety of indications including cancer, asthma, inflammation, heart failure, colitis, ischemia, and osteoporosis [2–5]. Selective EP_4_ modulators have been sought to provide the benefits of target modulation while limiting side effects arising from the modulation of the other receptor subtypes. EP_4_ is coupled to G protein-dependent pathways through G_αs_ and G_αi_, modulating adenylate cyclase activity and synthesis of intracellular cAMP. In addition to signaling through cAMP, biased agonism has been shown to play a role in the EP_4_ downstream signaling through the β-catenin and β-arrestin pathways [3, 6, 7].

GlaxoSmithKline, Roche Holding AG, Ono Pharmaceuticals and Merck and Co. have targeted EP_4_ with γ-lactam SAR programs [3, 8]. Multiple industrial drug-discovery programs have utilized the rat EP_4_ (rEP_4_) receptor as a surrogate for the human EP_4_ (hEP_4_) receptor in screening assays and animal models to identify lead compounds for development [9–15]. Potent, selective γ-lactam small molecule PGE_2_ mimic EP_4_ agonists were discovered using these screens and have advanced to various stages of preclinical and clinical development for several indications [9, 16]. Elimination of the stereocenter at the α-chain has been shown to be tolerated, beginning first with the discovery of 8-aza-11-deoxy-PGE_1_ [17]. Highly selective, potent agonists such as Roche 31 (CAY10684) have been reported [16]. Likewise, the role of agonists such as Rivenprost (Ono-4819) within bone health has been well established [14]. Additionally, full and partial agonists of differing chemical classes have been identified, including GSK’s reporting of non-prostanoid selective EP_4_ agonists [18]. Within the classes of prostanoid agonists, species selectivity and potency differences have been noted, including Sulprostone and Misoprostol, where both show significantly increased potency for the human EP_4_ receptor over rat receptor [19]. There is no reported mechanism driving the species difference between the rat and human receptors.

No known x-ray crystal structure of EP_4_ exists from any species. Mutational studies of hEP_4_ have implicated the hydroxyl of T168 as critical for PGE_2_ binding [20]. While currently no experimental structural data for EP_4_ exists, threading and docking studies have been performed with the hEP_4_ receptor. That study implicates a set of polar contacts comprising a binding motif of Y186^(5.40)^, F191^(5.45)^, S103^(3.41)^, S285^(6.48)^, D311^(7.35)^ [21]. Since publication of these studies, threading methodologies have advanced where solvation models have been improved [22, 23]. With the aim of better understanding the binding event of PGE_2_, evaluating the differences between the hEP_4_ and rEP_4_ receptors and to enable in silico drug discovery efforts, a homology modeling and docking study was undertaken focusing on these receptors. These models have been thoroughly validated by comparisons to binding and activation data.

## Results

**Fig. 1:**
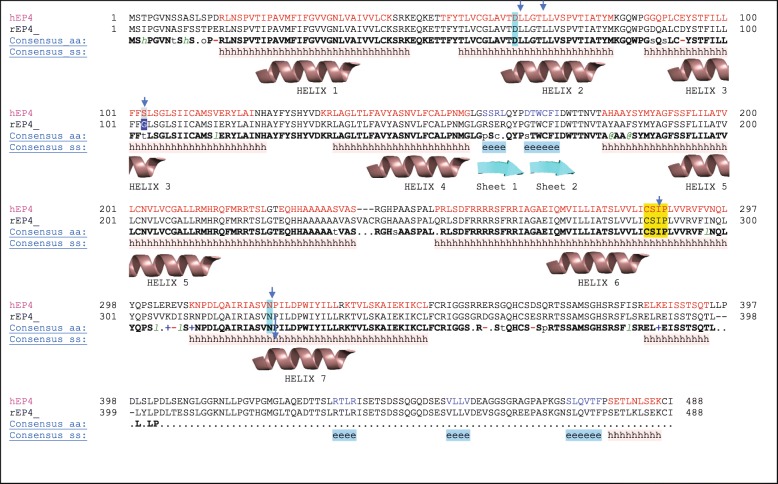
PBLAST alignment of the rEP_4_ and hEP_4_ receptors featuring the consensus sequence and secondary structural features as determined by Expresso TCoffee. Helix sequences are denoted by the letter h. Beta sheets are denoted by the letter e. The sequences are 88% identical and 92% homologous. The CSxP motif is highlighted in yellow. D65^(2.50)^ is highlighted in cyan, with an arrow pointing to the residue. Residue 103^(3.41)^ is marked with an arrow and highlighted in grey (hS103^(3.41)^) or blue (rG103^(3.41)^). Residue N ^(7.45)^ is highlighted in cyan, with an arrow pointing to the residue

**Fig. 2:**
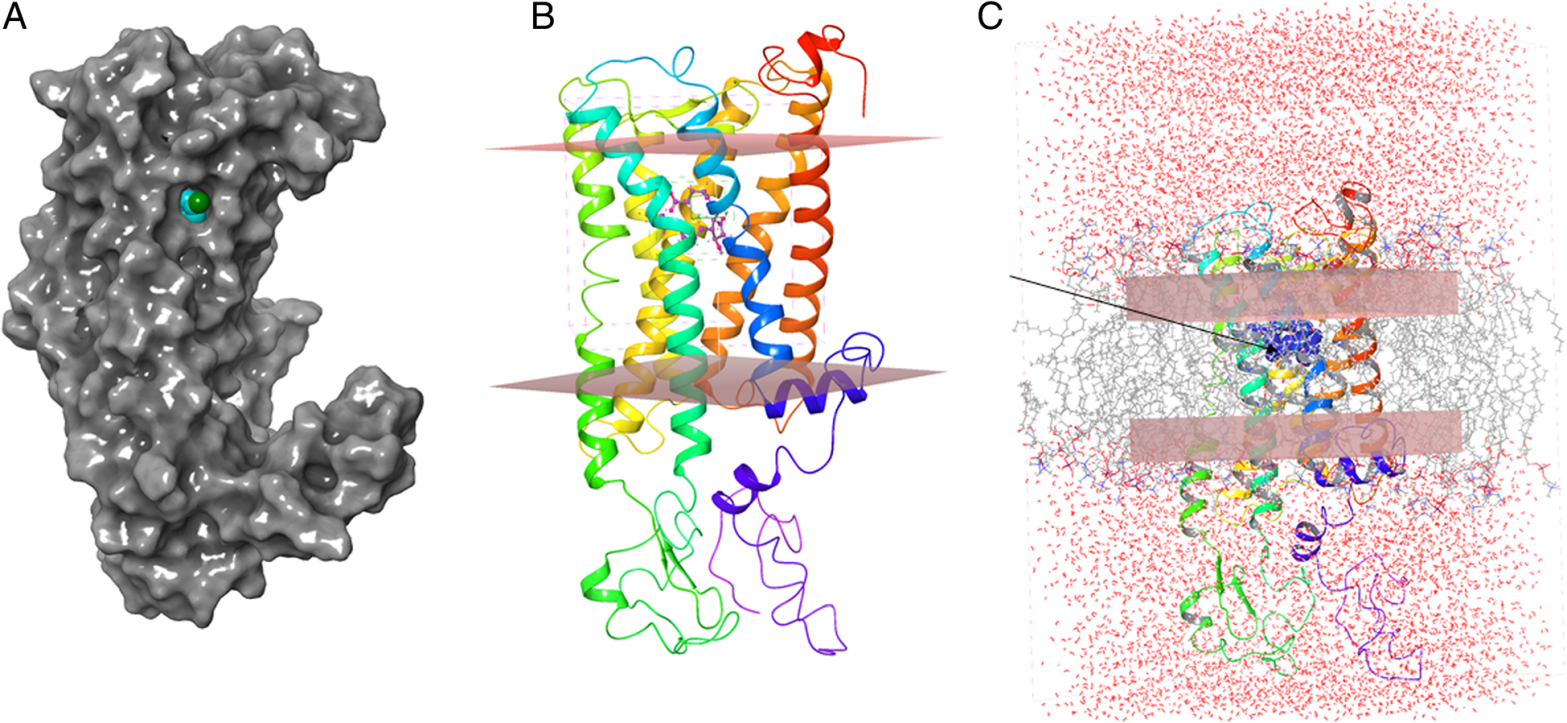
Representative images of rEP_4_ receptor with PGE_2_ docked. **a** Surface representation, highlighting channel between helix 5 and helix 6 (cyan/green surfaces), enabling interaction with molecules within phospholipid bilayer. **b** Edges of implicit membrane superimposed on cartoon representation of rEP_4_ receptor. **c** Desmond MD simulation model featuring explicit membrane. PGE_2_ shown in in blue with an arrow pointing to the site

**Fig. 3:**
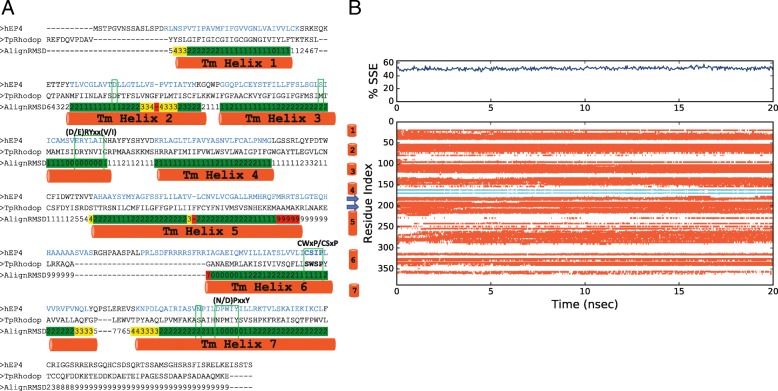
**a** Sequence alignment Squid Rhodopsin (PDB ID: 2ZIY) and hEP_4._ Structural annotations based on hEP_4_ model generated. RMSD alignment scores from RaptorX server used to score alignment within TM helical regions and colored from green (RMSD 0–2) to yellow (RMSD 3–4) to red (RMSD > 5). **b** Secondary structure stability by residue over time during 20 ns MD simulation

All reported models have been deposited to the ModelArchive (https://www.modelarchive.org/) [24, 25]. Sequence alignment and secondary structural prediction of the canonical 7 transmembrane helices are described in Fig. 1, as predicted using TCoffee Expresso Server [26, 27]. Between helices 4 and 5, a set of anti-parallel β-sheets form the cap of the receptors. Amino acid sequence alignment of the full-length rEP_4_ and hEP_4_ shows that they are 88% identical. Structural alignment of hEP_4_ and rEP_4_ models have an RMSD of 0.42 Å, suggesting differences between the proteins are subtle. The global structure of the human and rat EP_4_ homology models match well with the previously reported model (Fig. 2) [21]. A comparison of the hEP_4_ receptor homology models generated by Swiss-model and RaptorX is shown in Table 1. The ERRAT2 quality score, which is a measure of the quality of non-bonded interactions in comparison to high resolution structures, suggests that the RaptorX model of hEP_4_ was superior to the Swiss-model generated structure (Table 1) [28]. The model B-factor, a measure of uncertainty within the model, was larger within the RaptorX model. The Swiss-model featured loop deletions and truncations of the N-terminal and C-terminal regions of the receptor (regions of lower confidence) which improved the model B-factor. The Ramachandran plots from both models of hEP_4_ were similar (Table 1 & Additional file 1: Figure S1). Bond angle deviations and bond length deviations in both models were low (Table 1). Z-score, a measure of acceptable bond lengths, phi (φ) and psi (ψ) angles, and flags of distortions within planes, was used to identify geometry outliers. Within the hEP_4_ Swiss-model, Z-score outliers near the predicted binding site were identified including residues Y165 and Y188^(5.42)^ with scores of 19.66 and 19.10, respectively (Additional file 2: Figure S2). These geometric outliers near the reported binding site within the Swiss-model were of concern. Z-score outliers were identified within the hEP_4_ RaptorX model, including H129 (5.88), Y130 (7.55), were less significant compared to the Swiss-model structure (Additional file 2: Figure S2). Outliers within the rEP_4_ RaptorX model included F102 (11.49), H129 (8.22), H229-S234 (9.55, 10.09, 7.56, 7.56, 10.68, 5.02), A264 (10.49), and A265 (7.89) (data not shown). RaptorX model statistics are provided in full in (Additional file 9: Table S1). The model scores for the RaptorX hEP_4_ and rEP_4_ models were 269 and 268, respectively, where a score equaling sequence length is perfectly ideal (Additional file 9: Table S1). The uSeq percentages were nearly 60%, indicating a high confidence in the global structural fold (Additional file 9: Table S1). uGDT scores were > 100 for the RaptorX models with nGDT < 50, suggesting that specific regions of the model have increased uncertainty (SI Tale 1). The proposed binding sites are within the helical structure of the proteins, where the models provide the most confidence. An alignment of Squid Rhodopsin (PDB ID: 2ZIY) and RaptorX hEP_4_ model is shown in Fig. 3a with an alignment RMSD scoring function, displaying that the transmembrane helix regions are of high confidence [29]. The predicted transmembrane helical residues are denoted. Based on these results, the RaptorX models were used for follow-up study.

**Table 1:** Comparison of hEP_4_ models where the RaptorX model shows a better score for non-bonded interactions

	Swiss-model	RaptorX
B Factor (Å^2^)	0.578	63.223
Bond Angle Dev. (°)	2.118	2.242
Bond Length Dev. (Å)	0.015	0.013
Steric Clashes	0	1
Ramachandran Favored (%)	91.25	90.25
Ramachandran Allowed (%)	6.88	5.75
ERRAT2 Quality	69.677	87.056

The hEP_4_ model was stable over a 20 ns MD simulation, as seen in the secondary structural elements remaining stable through the simulation (Fig. 3b). The pose of PGE_2_ was stable over 20 ns, displaying an RMSD at or below 2Å throughout simulation (Additional file 3: Figure S3). Similarly, the rEP4 model was stable during a 20 ns MD simulation. For both models, the C_α_ root mean square fluctuation (RMSF) was between 1 and 2 Å for transmembrane regions and ligand contacts (Additional file 4: Figure S4). The sidechain RMSF was higher, but consistently between 1.5–2.5 Å for these same regions (Additional file 4: Figure S4). The most significant local structure movements during MD simulation between these models appear to occur at the intracellular loop between helix 5 and 6. During the simulation of hEP_4_, the RMSF spiked to 5 Å for residues 235–245, suggesting significant local movement within this range during the simulation (Additional file 4: Figure S4). The corresponding residues within rEP4 receptor displayed a slightly smaller movement of 4 Å (Additional file 4: Figure S4).

The substrate binding sites for these homologues are buried within the transmembrane region of the protein (Fig. 2b). The binding sites appear located closest to helices 5 and 6. When viewing the structures as a surface representation, both receptors show a narrow solvent channel potentially enabling interaction with the hydrophobic tails of the phospholipid bilayer, membrane dissolved lipids, or steroids (Fig. 2a). The structural alignment displays several differences within the predicted binding sites between hEP_4_ and rEP_4_, despite the high identity between the structures. A key active site residue S103^(3.41)^ in hEP_4_ (hS103^(3.41)^) is mutated to G103^(3.41)^ in rat (rG103^(3.41)^) (Fig. 1). Within helix 5, hS193^(5.47)^ aligns with rF193^(5.47)^, hY188^(5.42)^ with rA189^(5.42)^, and hL197^(5.51)^ with rA198^(5.51)^ (data not shown). Using SiteMap to predict the surface areas of the receptor binding sites, estimates the surface area of the human receptor to be 2174 Å^2^, whereas the rat receptor is predicted to be 1634 Å^2^ (data not shown) [30, 31].

**Fig. 4:**
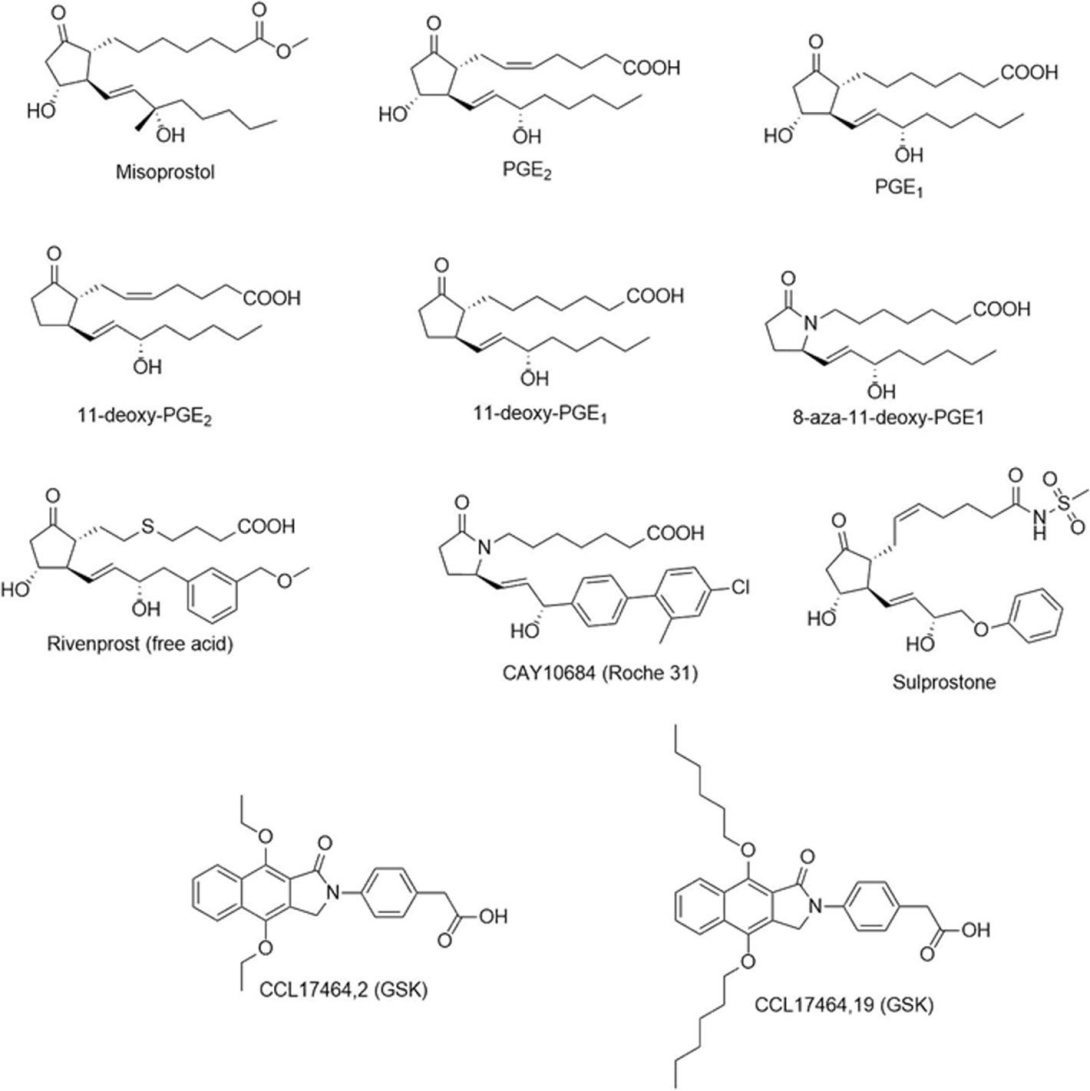
EP_4_ agonists chemical series

**Fig. 5:**
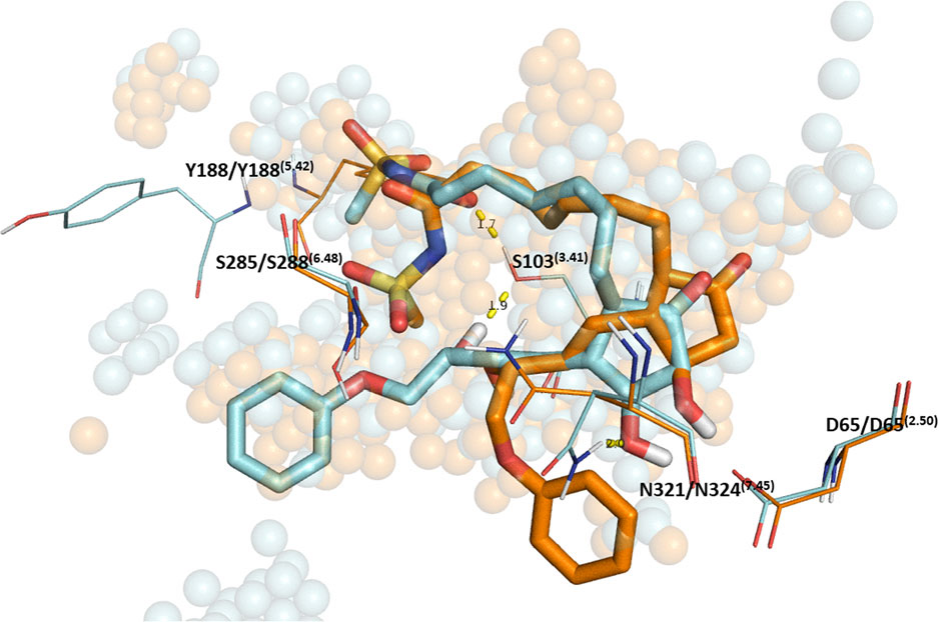
Docked pose of Sulprostone (orange) in stick representation overlaid within hEP_4_ receptor with versus docked pose within rat (teal). Sulprostone scored higher in human receptor and matches the pose of PGE_2_ within human receptor but fails to match this pose within rat receptor. Note interactions with S103^(3.41)^ within human model

To further validate the RaptorX models, several control experiments were performed. Previously reported data suggested that species specific differences were observed for the binding of two agonists, Sulprostone and Misoprostol [19]. These relatively weak agonists displayed greater binding within the human receptor versus the rat [19]. The structures of each are described in Fig. 4. Docking and scoring experiments properly rank-ordered the known agonists by docking score relative to each other and predicted the species-specific binding differences, more so in the case of Sulprostone (Table 2). The human model implicated S103^(3.41)^ with an H-bond to the N-(methylsulfonyl) formamide tail on the α-chain of Sulprostone, an interaction which was replaced with Y188^(5.42)^ within the rat model with an increase in distance. This observation supports the role of hS103^(3.41)^ to rG103^(3.41)^ in species-specific selectivity. MM-GBSA scoring shows a nearly − 20 kcal/mol difference in binding of Misoprostol and Sulprostone between the human and rat receptors, matching with the predicted species selectivity. A structural overlay of the docked poses is shown in Fig. 5.

**Table 2:**
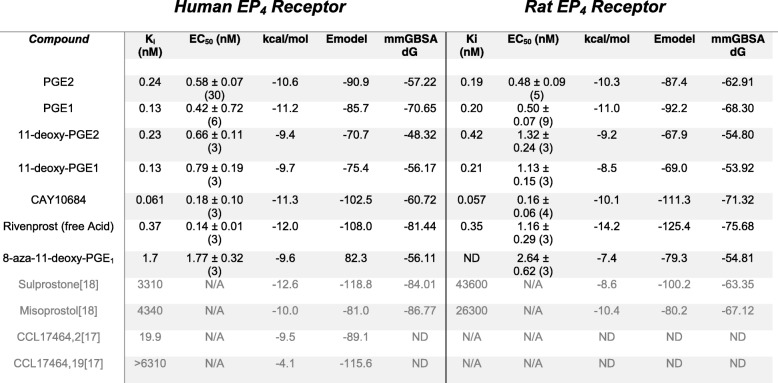
Direct binding affinities, activation effective concentrations, and docking scores for a series of EP4 agonists. K_i_ refers to equilibrium constant for the concentration of agonists to yield displacement of 50% of radiolabeled ligand. K_i_ = IC_50_/(1 ± [IL]/K_d_. EC_50_ refers to concentration where 50% activation of receptor within cAMP response assay is observed. Docking score is provided in terms of kcal/mol and is an empirical scoring of docked ligand affinity used for rank ordering. Emodel score can be used as a measure of confidence within the model. MMGBSA calculation is a force-field based method to score docked ligand affinity which correlated well with empirical scoring function. Values shown in grey were reported within cited references. N/A refers to data not available within literature. ND refers to data that was not determined within this study

**Fig. 6:**
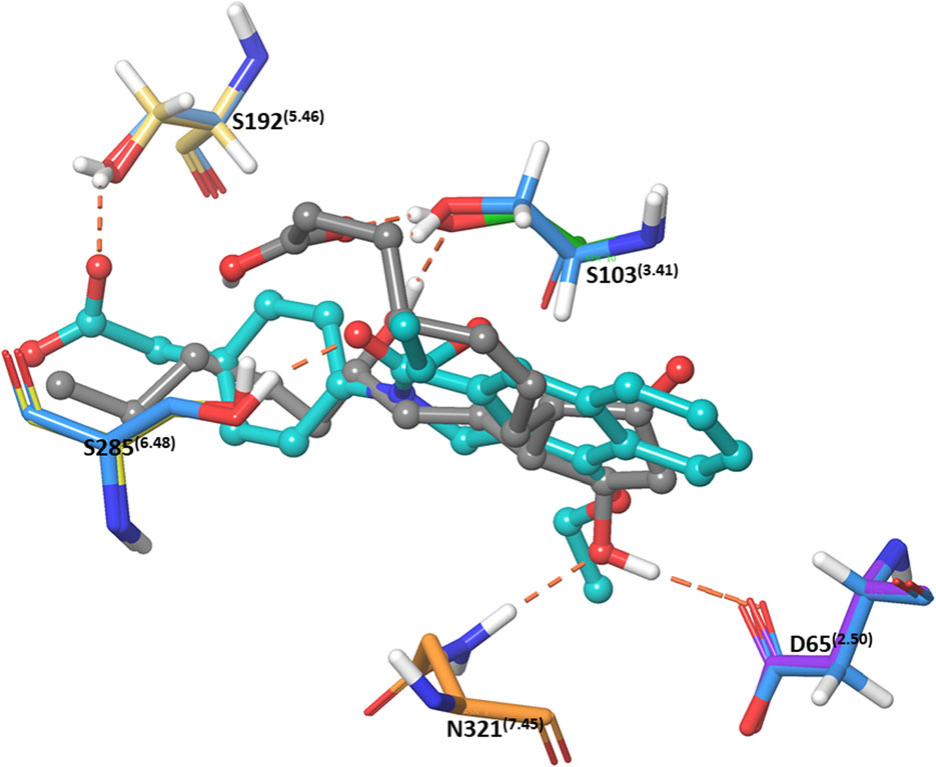
Docked pose of CCL17646,2 (teal) in stick representation overlaid within human EP_4_ receptor with versus docked pose of PGE_2_ (grey) within human EP_4_ receptor. Key residues are shown as line representations

GSK recently reported an SAR series with binding data for several non-prostanoid EP_4_ agonists [18]. The highest and lowest affinity compounds from this series corresponded to the non-prostanoid, fused benzoisoindol-1(3H)-one core with either a di-*p*-ethoxy substitution (CCL17464,2) or di-*p*-*n*-hexoxy substitution (CCL17464,19) (Fig. 4). These weak agonists were submitted for docking and scoring experiments and the model properly ranked-ordered the pairing within the hEP_4_ model which correlated well with reported binding affinities (Table 2). This suggested that the model could handle predictions using both prostanoid and non-prostanoid scaffolds. An overlay of the docked pose of CCL17464,2 versus the pose of PGE_2_ is shown in Fig. 6.

**Fig. 7:**
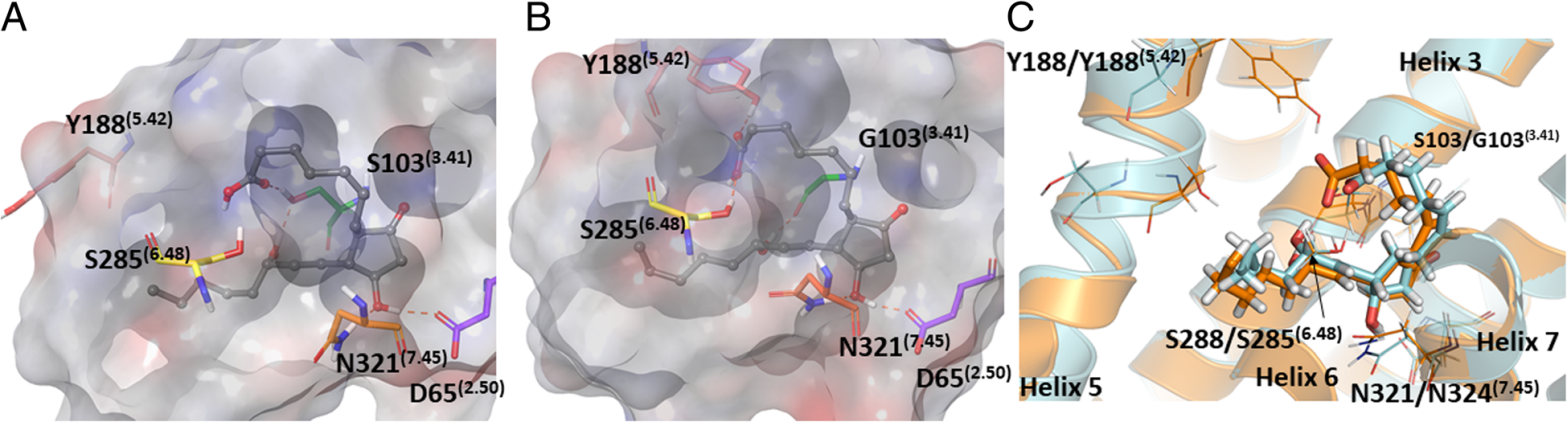
**a** Docked pose of PGE_2_ within hEP4 with key residues shown. **b** Docked pose of PGE_2_ within rEP_4_ with key residues shown. **c** Overlay of PGE_2_ docked into rEP_4_ RaptorX model (orange) aligned with PGE_2_ docked into hEP_4_ (teal). PGE_2_ is displayed as sticks, EP_4_ models are displayed as cartoons with key residues shown as line representations. The two poses align closely despite differences in predicted interactions

**Fig. 8:**
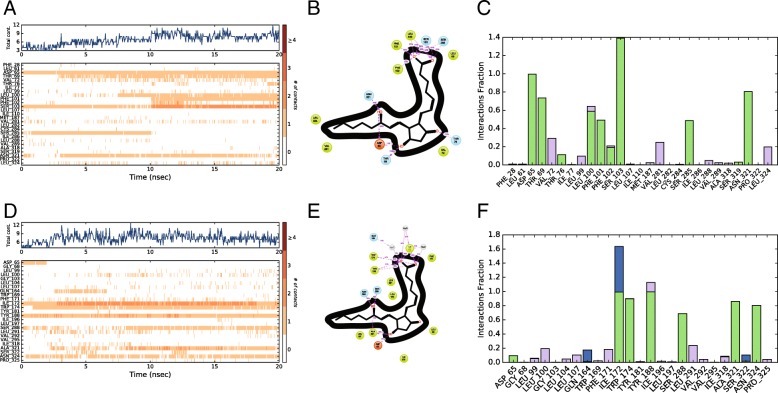
Desmond MD simulation using OPLS3 force field. Specific residue interactions are shown versus time. The overall favored interactions are shown in 2-D representation with the percent time during simulation where direct interactions are made. **a** Residue interactions with PGE_2_ within hEP_4_. **b** 2-D representation of PGE_2_ and its key interactions within human receptor. **c** Fraction of interactions captured between residues of hEP_4_ and PGE_2_, either through direct H-bonds (green), hydrophobic interactions (purple), water bridges (blue), or ionic contacts (none detected). **d** Residue interactions with PGE_2_ within rEP_4_. **e** 2-D representation of PGE_2_ and its key interactions within rEP_4_ receptor. **f** Fraction of interactions captured between residues of rEP_4_ and PGE_2_, either through direct H-bonds (green), hydrophobic interactions (purple), water bridges (blue), or ionic contacts (none detected)

Docking experiments were performed with a series of EP_4_ agonists of biological interest towards better understanding the binding of prostanoid agonists and to continue to validate our model. The prostanoid derived agonists in Fig. 4 were submitted for an induced fit docking experiment in both hEP_4_ and rEP_4_. A plot of Glide scores versus Emodel scores from a representative experiment is shown in Additional file 5: Figure S5. Poses with larger absolute values for both metrics were favored and selected as top poses. The highest scoring pose of PGE_2_ within hEP_4_, featured a hydrogen bonding network between the 15-hydroxyl of PGE_2_ in both the sidechain and backbone of S103^(3.41)^ (Fig. 7). S285^(6.48)^ sidechain serves as a hydrogen bond donor to the α-chain carboxylate (Fig. 7). The 11-hydroxyl of PGE_2_ also forms a hydrogen bonding network between N321^(7.45)^ and D65^(2.50)^ (Fig. 7). MD simulation shows a large amount of interactional flexibility within the binding event. D65^(2.50)^, S103^(3.41)^ serve as protein-ligand contact points throughout the entire simulation (Fig. 8). Polar contacts are exchanged through the simulation, D65^(2.50)^ makes contacts with 15-hydroxyl or 11-hydroxyl, N321^(7.45)^ makes contacts with 11-hydroxyl or 15-hydroxyl, S103^(3.41)^ makes contacts with carboxylate or 15-hydroxyl, and S285^(6.48)^ makes contacts with carboxylate or 15-hydroxyl. The α-chain carboxylate was observed to be highly flexible in its orientation, with a wide variety of torsions sampled. The radius of gyration, a measure of extendedness of a ligand and equivalent to its principal moment of inertia, hovered between 4.25 Å and 4.5 Å points to a stable orientation of the agonist within the binding pocket throughout the simulation (data not shown).

The residue specific interactions between PGE_2_ and the rEP_4_ receptor are different compared to hEP_4_, however the poses align nearly identically. Figure 7c features an overlay of the two structures displaying consistent binding poses despite which model was used. The rEP_4_ receptor makes polar contacts with PGE_2_ via residues Y188^(5.42)^, S288^(6.48)^, N324^(7.45)^, and D65^(2.50)^ (Fig. 7). The backbone interaction between the 15-hydroxyl-PGE_2_/rG103^(3.41)^ is less significant than the 15-hydroxyl-PGE_2_/hS103^(3.41)^ sidechain interaction. During MD simulation, the 11-hydroxyl of PGE_2_ relaxes and interacts with A321 backbone of rEP_4_ (Fig. 8). D65^(2.50)^ loses its direct interaction with the 11-hydroxyl of PGE_2_ after 2 ns (Fig. 8). S288^(6.48)^, N324^(7.45)^, I172, Y188^(5.42)^ make key interactions with PGE_2_ through entirety of simulation.

A set of known EP_4_ agonists were tested in radioligand binding and cAMP functional activation experiments. The scores from the docking experiment were compared with the functional data to better understand the model predictions (Table 2). Endogenous EP_4_ agonists are largely equivalent in both affinity and functional activation within the series with respect to their K_i_ and EC_50_ values for either the rEP_4_ or hEP_4_ receptor, suggesting little species-specific differences in the series_._ Between the receptors, ligands such as PGE_1_, 11-deoxy-PGE_2_, and PGE_2_ displayed a 0.2–0.3 kcal/mol difference with minimal preference for the human receptor (Table 2).

**Fig. 9:**
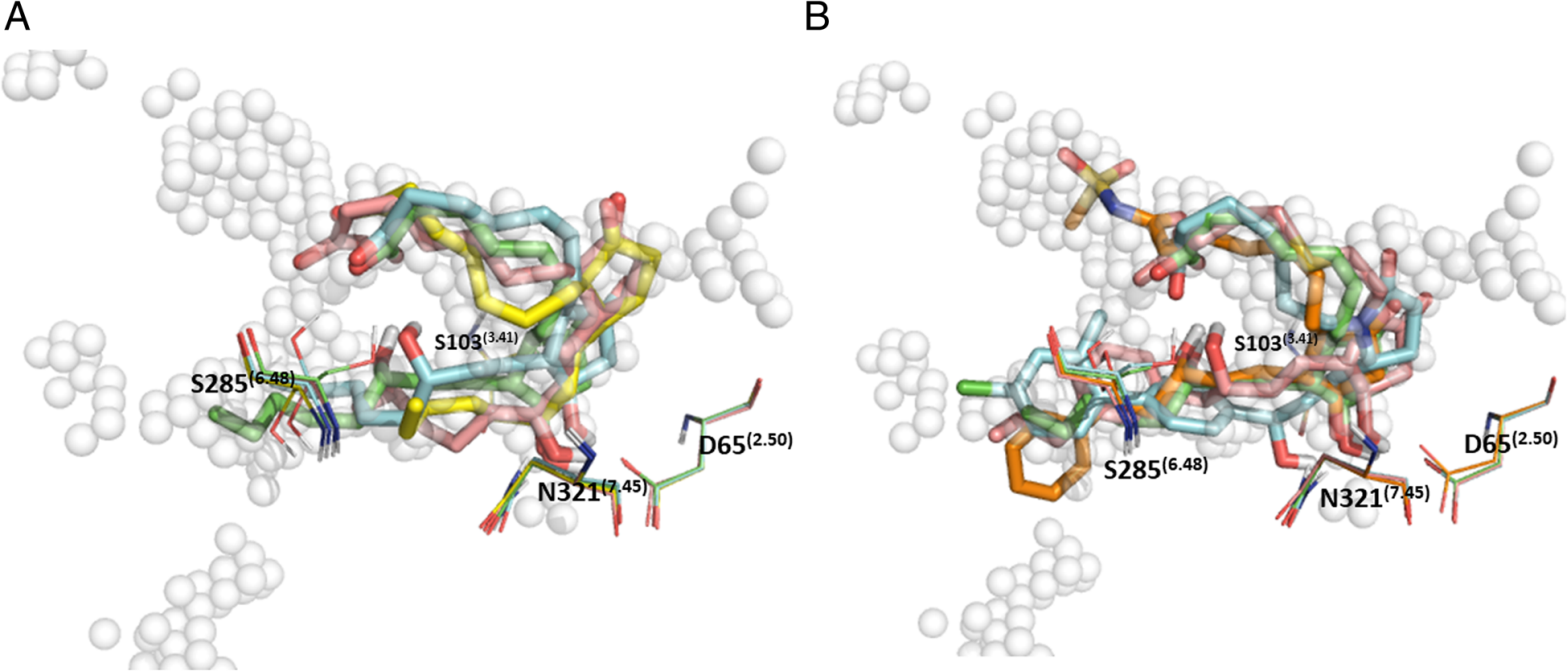
**a** Overlay of endogenous ligands PGE_2_ (green), PGE_1_ (teal), 11-deoxy-PGE_1_ (pink), 11-deoxy-PGE_2_ (yellow) docked on hEP_4_ receptor. SiteMap hot spot points shown as spheres, indicating shape of binding pocket. **b** Overlay of lead agonists Sulprostone (orange) CAY10684 (teal), Rivenprost (pink), PGE2 (green) docked on hEP_4_ receptor. SiteMap hot spot points are shown as spheres which indicate the shape of the binding pocket

Within the hEP_4_ model, the top scored pose of each agonist was structurally aligned and displayed in Fig. 9. D65^(2.50)^ H-bonding with γ-lactam hydroxyl is observed in most poses with N321^(7.45)^ H-bonding with the 15-hydroxyl. Overall, poses are consistent with the hEP_4_ model of PGE_2_ docking (Fig. 7). The shape of the predicted hEP_4_ binding site generated by SiteMap matches well with the poses [30, 31]. The CCL17464,2 pose compared to PGE_2_ overlays closely (Fig. 6), despite the different scaffolds. The model’s predictions appear to align better with the functional activation data where PGE_1_ and PGE_2_ trend towards better potency compared to their 11-deoxy counterparts (Table 2). We observed improved scores for both PGE_2_ and PGE_1_ over 11-deoxy-PGE_2_ and 11-deoxy-PGE_1_ within the model, matching the trend seen with the activation data (Table 2). Likewise, Rivenprost was the top docked compound and is the most potent agonist within the functional activation experiment (Table 2). The model highly favors interaction between D65^(2.50)^ and the γ-lactam 11-hydroxyl of these agonists. CAY10684 is among the most potent compounds tested in the human cAMP functional activation assay and was among the highest scoring compounds within the human model.

Rivenprost displays a binding mode similar to PGE_2_ with D65^(2.50)^ H-bonding with γ-lactam hydroxyl and N321^(7.45)^ H-bonding with the 15-hydroxyl. Rivenprost is the only compound which is significantly more potent in the functional activation assay by Unpaired T-test with two-tailed *p* value (*p* < 0.0001) and is the top scoring compound within this series, additionally supporting the notion that our model correlates well with the functional activation data determined within this study (Table 2). The α-carboxylate of the Rivenprost free acid interacts with S103^(3.41)^ and S285^(6.48)^. CAY10684 is observed to be an especially tight binder in the radioligand binding assay. In the modeled pose, its primary interaction is through a hydrogen-anion interaction between the α-carboxylate and the partially positive hydrogens of S103^(3.41)^ and S285^(6.48)^.

Molecular Mechanics/Generalized Born Surface Area ΔG (MM-GBSA) calculations were performed on docked poses of known EP_4_ agonists (Table 2). This scoring function ranked Rivenprost and CAY10684 among the highest predicted affinity molecules within the subset of known EP4 agonists, matching well with the cAMP activation and binding data for these molecules.

## Discussion

The homology models of hEP_4_ and rEP_4_ presented in this study represent an improvement upon the Margan model of hEP_4_ [21]. The RaptorX homology model was compared to a Swiss-model structure to directly associate our model versus the same homology modeling techniques used within the Margan study. Overall, our metrics for non-bonded interactions were favored, the global fold was identical, and we observed less significant Z-score outliers. The homology models presented within this study were minimized within an implicit membrane system using the OPLS3 force field while the Margan model used the OPLS2005 force field without a membrane calculation. The Margan model is not available on any public archives for the use of other research groups, whereas our data is available publicly. Margan referenced site-directed mutagenesis experiments, which identified several key residues for PGE_2_ binding including T199^(5.53)^, S278^(6.41)^, S285^(6.48)^, and R316^(7.37)^ [21]. This study’s hEP_4_ receptor docking experiments implicate S285^(6.48)^ through direct H-bond formation to docked PGE_2_. T199^(5.53)^, S278^(6.41)^ and R316^(7.37)^ are additionally located within 5 Å of the docked PGE_2_ and may play a role within extended H-bonding networks. Together the top pose matches well with the previously reported biological data. Both the Margan study and ours implicate S285^(6.48)^ and S103^(3.41)^ as key PGE_2_ contacts within hEP_4_. Our study implicates several transmembrane helix 2 residues including D65^(2.50)^ and T69^2.54^ as polar contacts interacting with 11-hydroxyl of PGE_2_.

The poses identified within this study are different than the pose proposed by the Margan model (Additional file 6: Figure S6) [21]. The top poses within this study appear to be deeper within the transmembrane helices region of the receptor. In the Margan model, Y186^(5.40)^ and F191^(5.45)^ are shown oriented inwards toward the binding pocket. With the improved solvation of our model, relaxed within an implicit membrane system, these residues are pointed towards the lipid facing inner membrane region and do not play a significant role in contacting the docked molecules. In agreement with the Margan model, we observe both S285^(6.48)^ and S103^(3.41)^ playing a key role interacting with the 15-hydroxyl of PGE_2_.

Structural comparison of hEP_4_ with the homology model of the human DP prostaglandin receptor bound to an antagonist, displays a RMSD of 4.6 Å [32]. Our binding site aligns with the 3–4–5-6 helix binding site and differs in the reported 1–2-7 antagonist binding site [32]. Our agonists dock closely to the CSxP motif of helix 6, where S285^(6.48)^ sidechain is 1.8 Å from the α-carboxylate of PGE_2_. This motif aligns with the CWxP motif within rhodopsin and other class A GPCRs, where it has been implicated as activation transmission switch [33, 34]. During review of this manuscript, a set of two crystal structures of the Human prostaglandin D2 receptor CRTH2 were released within the Protein Data Bank, although no publication has been yet to be published. These were crystallized with two antagonists bound, fevipiprant (PDB ID: 6D26) and CAY10471 (PDB ID: 6D27). These antagonists bind within the canonical 1–2-7 antagonist binding site, near T168. Structural alignment of the C(S/W) xP motif, shows that the predicted hEP_4_ S285^(6.48)^ is well oriented overlaying with W of the CWxP motif. Likewise, D65^(2.50)^ overlays well between the models. S103^(3.41)^ of the hEP_4_ structure is closely positioned to either a S118 or M115 residue of DP2 receptor (Additional file 7: Figure S7). Lastly, the crystal structures of human prostaglandin D2 receptor show Y188^(5.42)^ and Y186^(5.40)^ structurally aligning with hydrophobic residues (A205, F211, F215) largely oriented toward the hydrophobic inner-membrane space, and not towards the active site as seen within the Margan model [21].

Previous work identified T168 within the second extracellular loop of hEP_4_ as being essential for PGE_2_ binding [20]. This residue is observed at the turn of the anti-parallel β-sheets which serve as the cap of the receptor within this model. This is located within the identified antagonist binding site, as seen in DP2 receptor, and likely plays a key role in receptor conformation. T168 is observed making a hydrogen bond with Y80^(2.65)^, which may be essential for orienting the beta-turn-beta and control of TM helix 2 orientation.

Experimental radioligand competition data and functional activation data was collected within this study providing additional datasets that align with the homology models. The [H^3^]-PGE_2_ binding results are consistent with binding data reported previously such as PGE_2_ being measured at a K_i_ of 0.72 nM against hEP_4_ previously and measured as 0.58 nM within this study [19, 35]. The models appear to align better with the rank order of the cAMP activation data versus the competitive binding data. This result may be a consequence of the use of an empirical scoring function for rank ordering. MM-GBSA scores were also determined (Table 2). This is a force-field based method to estimate the free energy of binding by estimating the energy of the complex versus the free receptor and ligand. The rank-order from MM-GBSA did not always align with the empirical scoring, implying this result may be an artifact of the compounds selected in conjunction with the inherent error in the model’s predictions. Lastly, the statistical power behind the radioligand competition experiments was insufficient to determine the significance of those values. Thus, differences between certain compounds may be insignificant.

The model performed much better at predicting the species-specific differences within Sulprostone compared to Misoprostol, when using empirical scoring. This may partly be due to the limited structural differences between Misoprostol and the endogenous EP_4_ agonists, where the model fails to capture the significance of 15-methyl in terms of affinity differences. However, using MM-GBSA calculation, the predicted binding difference between the receptors is more significant and the species selectivity is more obvious. Surprisingly, being agonists with μM affinities and primary selectivity for EP_1_ & EP_3_, the compounds unexpectedly scored well by both empirical scoring and force-field. As the direct binding results for Misoprostol and Sulprostone are presented from the literature, it is unclear if differences within methodologies play a role in this discrepancy. Likewise, this observation points to the inherent limitations to the use of a homology model which likely fails to capture receptor subtype differences that exist between hEP_4_, hEP_3_, hEP_2_, and hEP_1_. Of the compounds tested, Rivenprost appears to display the most species-specific selectivity within activation of the EP_4_ receptor. For receptor activation, the EC_50_ was measured to be 0.142 nM and 1.16 nM for hEP_4_ and rEP_4_, respectively (Table 2). The radioligand experiments estimated the K_i_s to be 0.37 nM and 0.35 nM for hEP_4_ and rEP_4_, respectively. MM-GBSA predicts a 5.76 kcal/mol difference in binding to the receptors, suggesting it may support that there is species-selectivity. The model predicts a species difference for CAY10684 with a 10.6 kcal/mol difference, an effect not observed within the in vitro functional activation or direct binding studies.

These models provide a resource to enable in silico drug discovery and allow for the design of mutational studies to further understand the binding of PGE_2_ and EP_4_ activation. Mutational studies should be performed to validate these models and their predictions, including G103S in rEP_4_, to test if this ablates species-selectivity. The mutations D65A, N324A, S288A, Y188F would be useful for confirming the essential nature of these predicted interactions within rEP_4_. Likewise, the mutations D65A, S103G, S103A, S285A, N321A would be informative for confirming the essential nature of these predicted interactions within hEP_4_. This model can also be used by researchers for in silico alanine scanning to assist in their predictions of binding interactions between these receptors with novel scaffolds. In the absence of an available crystal structure, these models will allow for hypothesis generation for researchers within the field of prostaglandin biology. Work is on-going to enhance these models including utilizing the data from the prostaglandin D2 receptor to refine these models.

## Conclusions

This work has generated a set of novel homology models of the rEP_4_ and hEP_4_ receptors. The models provide an improvement upon the previously reported structure, largely due to improved solvation by use of an implicit membrane model. Structural statistics and biological data compared to this model support their validity and highlight their limitations. The hEP_4_ empirical scoring model appeared to align best with the cAMP activation data in rank order of agonist potency. This rank ordering matches closely with the rEP_4_ receptor. The model also successfully rank-ordered two compounds from a previously reported SAR series where the steric bulk of a para substituted di-*p-n*-hexoxy on a non-prostanoid heterocyclic core was disfavored over the di-*p*-ethoxy [18]. Likewise, species-specific differences between these models were highlighted and a test series of known agonists with reported species-selectivity were screened against each model. In both cases, the weak agonists Sulprostone and Misoprostol were known to bind with higher affinity to the human structure [19]. The models generated scores that were substantially higher for the human receptor versus rat for Sulprostone and Misoprostol. The radioligand competition and function activation data presented within this study are similar to the previously reported data. Our agonists dock closely to the CSxP motif of helix 6, where S285^(6.48)^ sidechain is 1.8 Å from the α-carboxylate of PGE_2_. This motif aligns with the CWxP motif within rhodopsin and other class A GPCRs, where it has been implicated as activation transmission switch [33, 34]. These structures are a significant advancement compared to the Margan model and enable in silico drug discovery efforts targeting the EP_4_ receptor.

### Addendum

The work presented within this study using the native and unbiased form of hEP_4_, was performed completely independently from the crystal structures of hEP_3_ (PDB IDs: 6M9T & 6AK3) and hEP_4_ (PDB IDs: 5YHL & 5YWY) [36–39]. Both crystal structures were released on December 5th, 2018, while this manuscript was still in review following our initial submission on June 15th, 2018. Our revised manuscript following reviewers’ edits was resubmitted on November 25th, 2018. This addendum was added March 29th, 2019 following final revisions to highlight some features of the crystallographic data.

While the first crystal structure of hEP_4_ represents a major milestone for understanding the biology of prostaglandins, the use of homology modeling for this target is still necessary. The published hEP_4_ crystal structure used an altered form of the receptor (via thermostable mutations) which stabilized the receptor in its inactive conformation [36]. These mutations and the addition of a bound Fab molecule weighted the receptor toward the inactive form and thus enhancing antagonist binding [36]. Previously, the adenosine receptor A2a had been shown to have a Na^+^ binding pocket with proposed importance to receptor activation [40]. The thermostable mutation G106R^3.39^ presented within the hEP_4_ crystal structure likely disrupted this pocket again forcing the structure into an inactive form [36]. As a result, the need for improved models of the active conformation of hEP_4_ remains.

The active conformation of the hEP_3_ PGE_2_-bound structure shows the ω-chain of PGE_2_ oriented toward W295^(6.48)^ of the CWxP motif making a direct interaction with D99^(2.50)^ of the reported Na^+^ binding pocket [38]. Within the homology model presented in this work, direct interactions to the corresponding S285^(6.48)^ of the CSxP motif and D65^(2.50)^ are predicted. As the Na^+^ pocket may serve as a negative regulator, *Morimoto* et al *2018* suggested this may be the mechanism for receptor activation [38, 40].

Several residues that make significant interactions with the ONO-AE3–208 ligand in hEP_4_ are also highlighted by our docking predictions including L99^(3.31)^, T76^(2.61)^, V72^2.61^, W169^(ECL2)^ which largely form a hydrophobic pocket near the E ring of PGE_2_ [36]. Our model failed to identify Y80/Y114^(2.65)^, a key contact for the alpha-chain carboxylate observed in both the hEP_3_ and hEP_4_ crystal structures as a “key” ligand contact. This manuscript, however, did note the potential importance of this residue independently: “T168 is observed making a hydrogen bond with Y80^(2.65)^, which may be essential for orienting the beta-turn-beta and control of TM helix 2 orientation” (Discussion Section). Additionally, our model also predicts the importance of S103^(3.41)^, a prediction which requires additional biochemical or structural information to confirm. Similarly, our model highlights the importance of residues within the putative Na^+^ binding pocket including S285^(6.48)^ and D65^(2.50)^. We have added a (Additional file 8: Figure S8) showing the induced fit docking of PGE_2_ within a homology model based on the hEP4 crystal structure [36]. Interactions between Y80^(2.65)^ and R316^(7.40)^ with the alpha chain carboxylate were observed. The PGE_2_ 11-hydroxyl makes hydrogen bond with hEP_4_ S69^(2.54)^ side chain. The carbonyl oxygen appears to also contact T76^(2.61)^. The PGE_2_ 15-hydroxyl is near D65^(2.50)^ (4.3 Å) and is close to creating D65^(2.50)^ backbone interactions. In summary, both corroborating and contradictory data presented in this manuscript, supports many of the contacts highlighted in both the hEP_3_ and hEP_4_ crystal structures and continues to solidify both the PGE_2_-D65 interaction and putative Na^+^ binding pocket which plays a key role in agonist binding and ultimately receptor activation.

## Methods

### Receptor homology modeling

The sequences for *R. norvegicus* EP_4_ (rEP_4_) receptor and *H. sapiens* EP_4_ (hEP_4_) receptor obtained from National Center for Biotechnology Information (NCBI) using accession codes NP_114465.3 and NP_000949.1, respectively. These were submitted to RaptorX structure prediction suite (http://RaptorX.uchicago.edu/) and analyzed to confirm the quality of the model using the *P*-value, global distance test, absolute global quality, and RMSD [23, 41–46]. RaptorX utilized squid and human rhodopsin as the primary templates for both rat and human EP_4_ threading. A full list of templates is provided in the supplemental. Sequences were additionally submitted to Swiss-model through the ExPAsy molecular biology suite (https://swissmodel.expasy.org) to provide additional models for global comparison. Models were prepared for docking using the protein preparation wizard within Schrödinger Maestro 11, adding hydrogens, creating disulfide bonds, and generating protonation states using Epik at pH 7.0 ± 2.0 [47]. H-bond assignments and torsion angles were optimized using PROPKA and a restrained minimization was performed using OPLS3 force field [48–51]. Loop refinement was performed in the presence of an implicit membrane. Membrane width was estimated by submitting to OPM Membrane Server (http://opm.phar.umich.edu/server.php) with values of 30.6 Å for rat EP_4_ receptor and 27.5 Å for human EP_4_ receptor [52]. Orientation of implicit membrane was completed using the global structure excluding loop residues (1–18, 295–309, 364–402) within the atom specification language (ASL).

### Induced fit docking with Schrödinger maestro

All ligand files were prepared and generated within Maestro 11. Ligands were prepared with LIGPREP from SMILES using OPLS3 force field modified using EPIK [22, 49–51, 53, 54]. Molecules were desalted and featured all possible tautomers. Binding site detection was performed using SiteMap with 20 site points required per reported site, using a fine grid, and using a more restrictive definition of hydrophobicity [30, 31]. Initial rigid body docking of PGE_2_ was used to center the box for a more extensive Induced Fit docking experiment [47, 55–58]. The glide grid was centered on residues previously identified as being near the binding site including S103^(3.41)^, S285^(6.48)^, S319 and G103^(3.41)^, S288^(6.48)^ for human and rat receptors, respectively. Extra precision (XP) protocol was used in Glide with Van der Waals scaling factor set to 1.0 and a partial charge cutoff of 0.15. Following Glide docking, the receptor and top scoring pose were merged and the ligand was selected to center the grid box for induced fit docking [58]. A search grid of 8,000 Å^3^ was centered on the docked PGE_2_. Residues within 5 Å of glide poses were refined using Prime with additional residues being refined as well including S103^(3.41)^, Y165, I172, Y184^(5.38)^, Y186^(5.40)^, F191^(5.45)^, L195^(5.49)^, S285^(6.48)^, D311^(7.35)^, S319^(7.43)^ and G103^(3.41)^, L107^(3.45)^, W175, Y181, Y188^(5.42)^, S185^(5.39)^, S193^(5.47)^, S288^(6.48)^ for the human and rat receptors, respectively. Prime refinement incorporated the implicit membrane prepared earlier. Glide redocking was performed within 30 kcal/mol of best structure and for top 20 structures overall using XP protocol. PGE_2_ poses were evaluated based on docking score, Emodel score, and GlideScore. Follow-up compounds were docked with the grid centered on the docked pose of PGE_2_ using the same settings as above.

### Molecular dynamics simulations

Molecular dynamics simulations were prepared with Desmond using the classical simulation system set-up [59]. TIP3P solvent model was implemented and the membrane was placed excluding disordered loops (residues 1–18, 295–309, 364–402) from ASL. The box volume was minimized automatically using an orthorhombic shape. Ions within 20 Å of agonist were excluded and the model was neutralized by the addition of Cl- ions from 0.15 M sodium chloride and solvated using an OLPS3 force field [22, 50, 51]. Simulations were performed for 20 ns with 500 frames. Temperature and pressure were 300 K and 1.01325 bar, respectively. Simulations were analyzed using Schrödinger Maestro Simulation Event Analysis wizard.

### EP_4_ receptor binding

Radioligand binding experiments were carried out versus the hEP_4_ and rEP_4_ receptors. Human EP_4_ receptor binding was originally determined by Eurofins Pharma Discovery Services (Bois-l’Évêque, France) and confirmed in our laboratory. We also implemented the rEP_4_ receptor binding studies. Equilibrium competition binding assays were conducted to determine the inhibition constants (Ki) for ligand interactions with EP_4_ receptors sourced from membranes (160,000×*g*) of HEK293 cells overexpressing recombinant hEP_4_ or rEP_4_ receptors. Ligand competitions were conducted from 30 nM to 30 pM against [H^3^]-PGE_2_ (180 Ci/mmol; NEN Radiochemicals, Boston, MA), which was held constant at 0.5 nM. Assays were performed in 10 mM 2-(N-morpholino) ethanesulphonic acid (MES) buffer containing 1 mM EDTA and 10 mM MnCl_2_, pH 6.0, during 120 min at room temperature. K_i_ was calculated using the Cheng Prusoff equation where K_i_ = IC_50_/(1 ± L/K_d_). A Scatchard plot was used to determine K_d_ for [H^3^]-PGE_2._ The calculated K_d_ for [H^3^]-PGE_2_ was 0.3 nM.

### EP_4_ receptor functional activation

Human and rat EP_4_ receptors were expressed in HEK293T/17 cells separately by reverse transfection on 96-well plates with optimized amounts of cDNA expression constructs immobilized on the surface. Activation of EP_4_ receptor in this cell line was measured as an increase in cAMP levels using an ELISA kit (Cayman Chemical, Ann Arbor MI). In short, the transfection plates were seeded with 50,000–80,000 cells/well in reduced serum medium containing 0.5% FBS and incubated overnight at 37 °C with 5% CO_2_. Before stimulation with test compounds, media was aspirated from the wells and replaced with reduced serum media containing 0.5 mM 3-isobutyl 1-methylxanthine (IBMX). Compound dilution series were prepared in the same IBMX containing media at 2X final concentration and applied to the wells in equal volume. Stimulation media were aspirated from the plates after 30 min incubation with test compounds, and the plates with cells were placed on dry ice and then put into − 80 °C freezer for > 2 h. Cell lysates were prepared on thawed plates by adding 200 μl/well ice-cold ELISA buffer with 0.05% Tween-20 followed by titration. The cell lysates were then transferred to a polypropylene plate and clarified by centrifugation at 1000×g for 15 min at 4 °C. Supernatant was transferred into a pre-coated mouse Anti-Rabbit IgG ELISA plate together with serial dilutions of cAMP for a standard curve in triplicate. cAMP-acetylcholinesterase conjugate (cAMP tracer) was added, followed by cAMP EIA antiserum. Plates were incubated overnight with shaking. After washing 5 times in washing buffer, Ellman’s reagent was added to the well and shake for 90 min under cover before measuring absorbance at 405 nm on a plate reader.

## Additional files

Additional file 1 : **Figure S1.** (A) Swiss-model Ramachandran plots for the hEP_4_ model. (B) RaptorX Ramachandran plots for the hEP_4_ model. (C) RaptorX Ramachandran plots for the rEP_4_ model. Plots were similar for hEP_4_ and rEP_4_ for the residues within favored regions (90.25%/88.09% of residues, human/rat) and allowed regions (5.75%/6.70%, human/rat). Swiss-model displayed improved Ramachandran plot versus RaptorX. (PNG 173 kb)

Additional file 2 : **Figure S2.** (A) Z-score outliers for hEP_4_ RaptorX model (B) Z-score outliers for hEP_4_ Swiss-model. Red lines indicate residues with Z-score warnings. Green corresponds to near ideal Z-scores. (PNG 133 kb)

Additional file 3 : **Figure S3.** MD simulation Ligand RMSD v. Time (20 ns) for (A) hEP_4_ and (B) rEP_4_. (PNG 91 kb)

Additional file 4 : **Figure S4.** MD simulation Protein RMSF v. residue (during 20 ns simulation) for (A) hEP_4_ and (B) rEP_4_. Smoothed curve over 5 neighboring residues to reduce noise. Red areas indicate alpha helical regions. Green lines indicate ligand contacts. Brown line indicates RMSF for sidechains. Blue line indicates Cα RMSF. Red line indicates B factor, shown only on hEP_4_ plot. (PNG 132 kb)

Additional file 5 : **Figure S5.** Representative image of Emodel scores versus Docking score for a set of agonists docked into hEP_4_. (PNG 77 kb)

Additional file 6 : **Figure S6.** Side by side comparison of the hEP_4_ docked PGE_2_ poses between the Margan model [20] and this study from the same angle. (PNG 525 kb)

Additional file 7 : **Figure S7.** Alignment of PGE_2_ docked to hEP_4_ with Crystal structure of the prostaglandin D2 receptor CRTH2 with fevipeprant. Top down view of receptor with transmembrane helices numbered. hEP_4_ receptor is display in rainbow coloring. DP2 receptor is shown in teal. Key residues for predicted hEP_4_ agonist binding site are shown with the corresponding DP2 residues overlaid. C(S/W) xP motif overlays between models with W of the CWxP motif overlaying with S285^(6.48)^. D65^(2.50)^ overlays well between the models. S103^(3.41)^ of the hEP_4_ structure is closely positioned to either a S118 or M115 residue of DP2 receptor. (PNG 1136 kb)

Additional file 8 : **Figure S8.** Homology model of the hEP_4_ receptor docked with PGE_2_. All 5 poses output from an induced fit protocol are shown [45, 53–56] . PGE2 is displayed in ball and stick representation with non-polar hydrogens hidden (gray). Dotted lines indicate hydrogen bonding interactions. hEP_4_-PGE_2_ interactions include: (1) The alpha chain carboxylate to both Y80^(2.65)^, and R316^(7.40)^ (2) The 11-hydroxyl to the OH group of S69^(2.54)^ (3) The carbonyl oxygen/T76^(2.61)^ and (4) The 15-hydroxyl to D65^(2.50)^ is 4.3 Å and within proximity to the hEP4 backbone. NOTE: This figure is part of the addendum and added to the original manuscript on March 29th, 2019. All other sections of this report were prepared prior to December 5th, 2018 release of EP_4_ crystal structure. (PNG 451 kb)

Additional file 9 : **Table S1.** RaptorX threading model statistics, before minimized refinement within Maestro. (DOCX 14 kb)

## Data Availability

All models reported here have been deposited within the ModelArchive (https://www.modelarchive.org/). hEP_4_ (DOI: 10.5452/ma-aasgt) & rEP_4_ (DOI: 10.5452/ma-aio8z).

## Abbreviations

COX: Cyclooxygenase
hEP_4_: human prostaglandin E2 receptor subtype 4
MM-GBSA: Molecular Mechanics-Generalized Born Surface Area
PGE_1_: Prostaglandin E1
PGE_2_: prostaglandin E2
rEP_4_: rat prostaglandin E2 receptor subtype 4
