# Supplementary figures and images for: Improved homology modeling of the human & rat EP4 prostanoid receptors
Source: BMC Mol Cell Biol. 2019 Aug 27;20:37. doi: 10.1186/s12860-019-0212-5 (PMC6712885; doi:10.1186/s12860-019-0212-5)

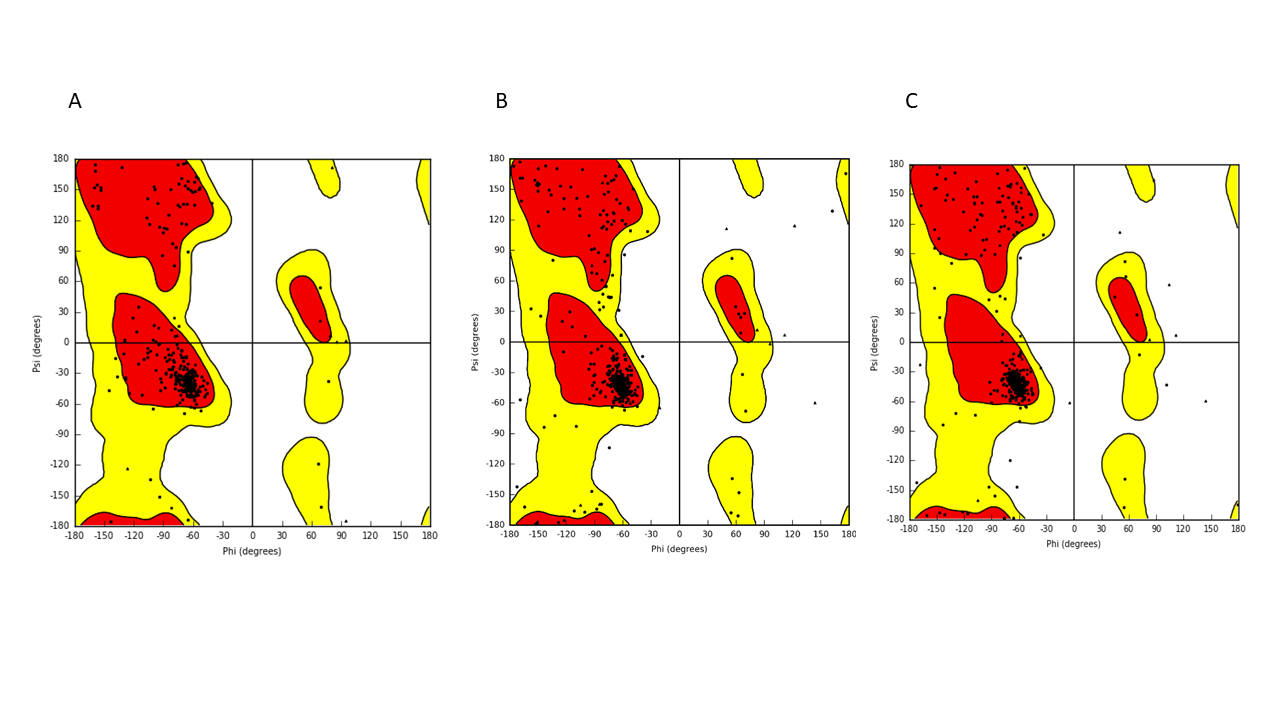

Supplement: Supplementary file 1 — : Figure S1. (A) Swiss-model Ramachandran plots for the hEP4 model. (B) RaptorX Ramachandran plots for the hEP4 model. (C) RaptorX Ramachandran plots for the rEP4 model. Plots were similar for hEP4 and rEP4 for the residues within favored regions (90.25%/88.09% of residues, human/rat) and allowed regions (5.75%/6.70%, human/rat). Swiss-model displayed improved Ramachandran plot versus RaptorX. (PNG 173 kb) [file 12860_2019_212_MOESM1_ESM.png]

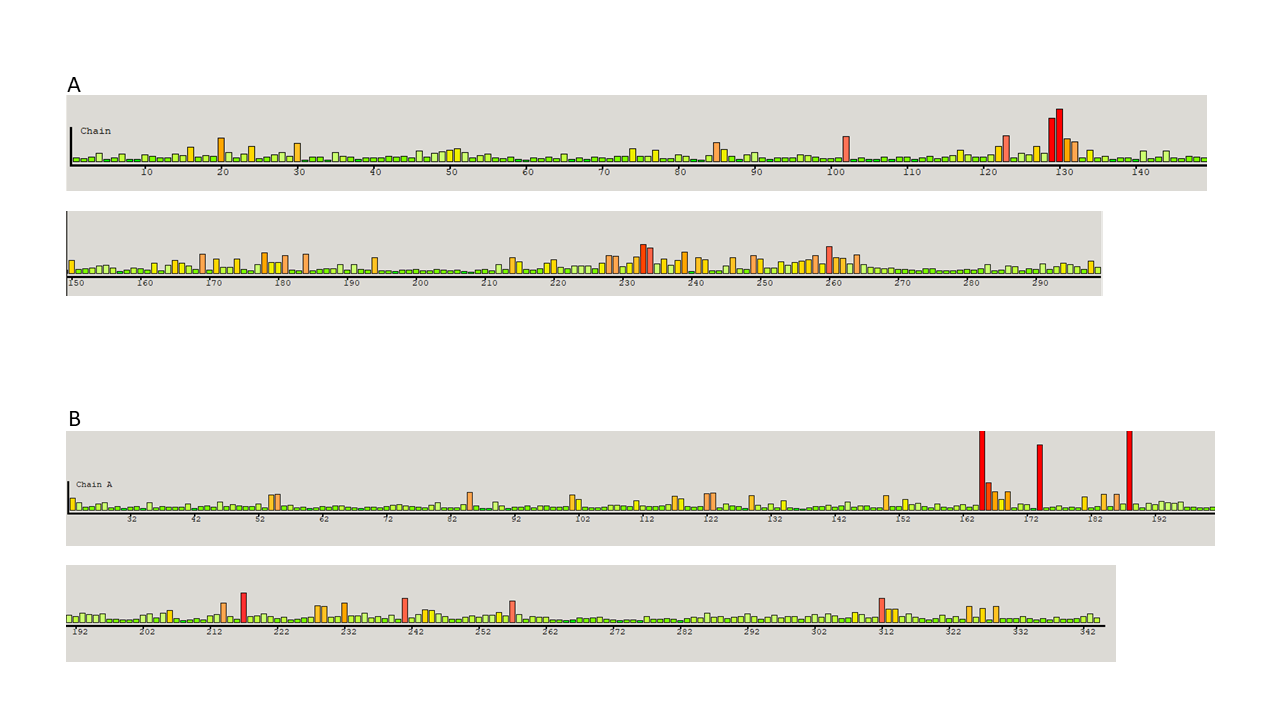

Supplement: Supplementary file 2 — : Figure S2. (A) Z-score outliers for hEP4 RaptorX model (B) Z-score outliers for hEP4 Swiss-model. Red lines indicate residues with Z-score warnings. Green corresponds to near ideal Z-scores. (PNG 133 kb) [file 12860_2019_212_MOESM2_ESM.png]

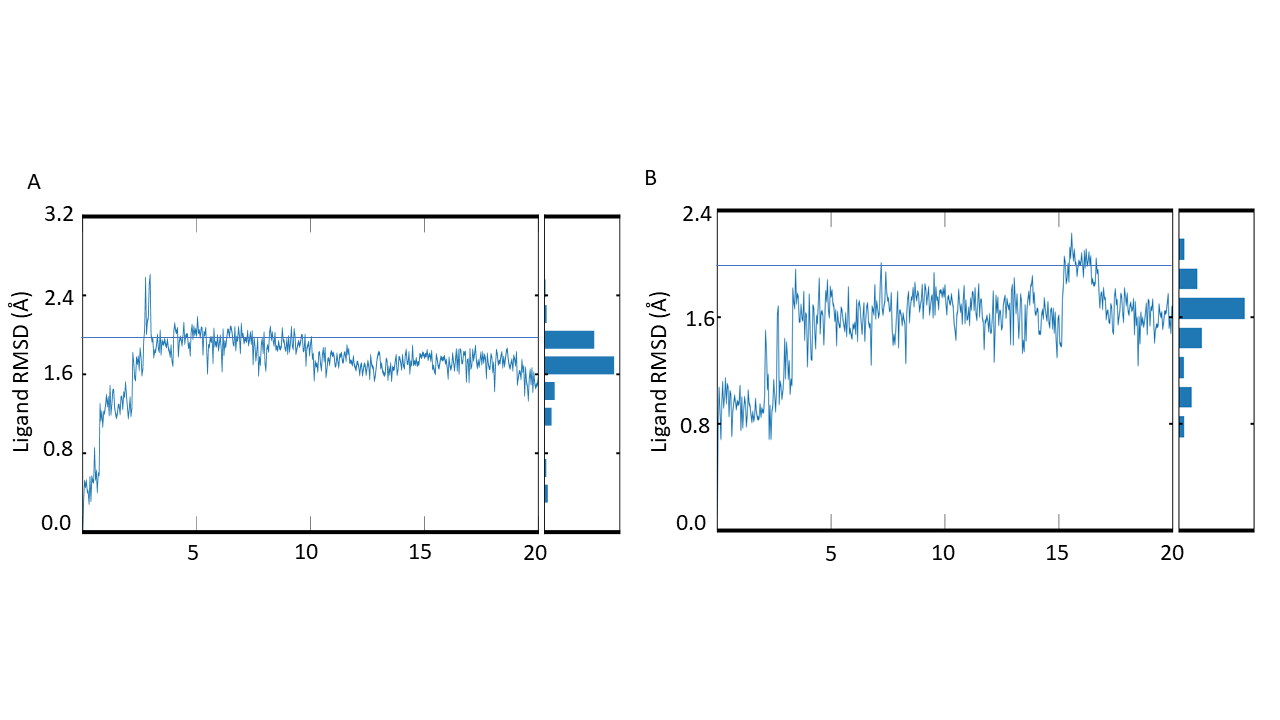

Supplement: Supplementary file 3 — : Figure S3. MD simulation Ligand RMSD v. Time (20 ns) for (A) hEP4 and (B) rEP4. (PNG 91 kb) [file 12860_2019_212_MOESM3_ESM.png]

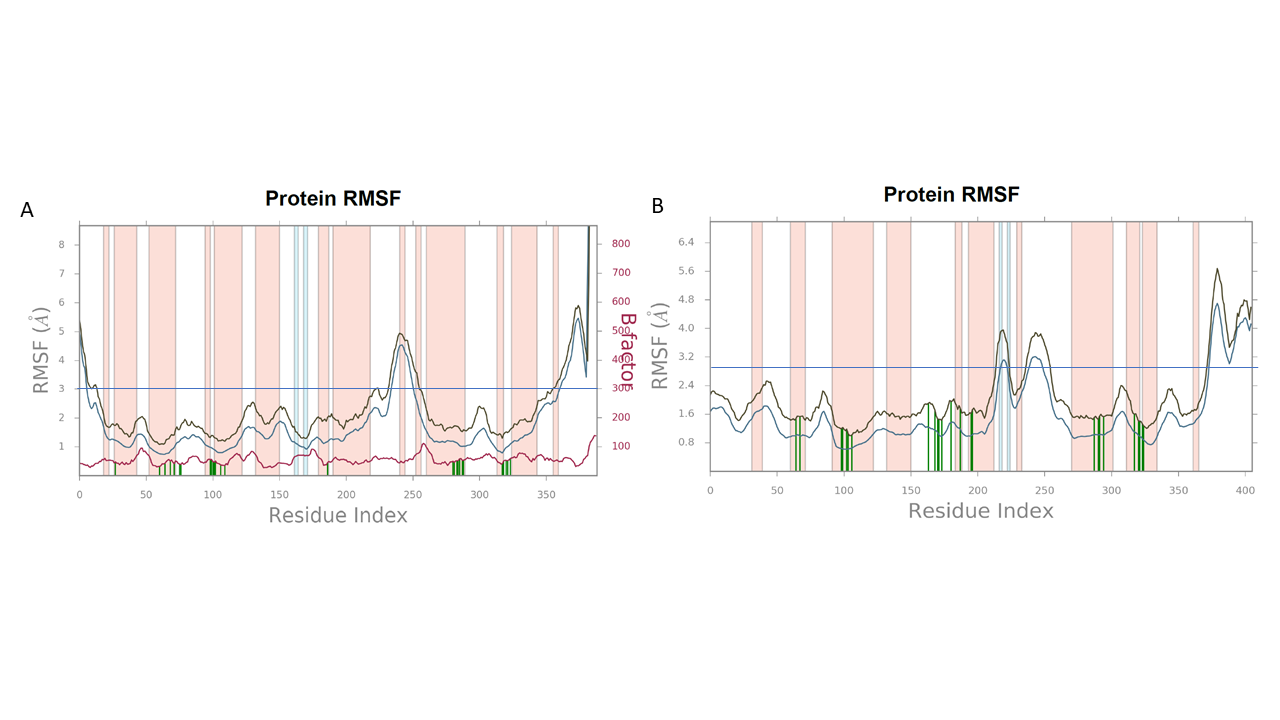

Supplement: Supplementary file 4 — : Figure S4. MD simulation Protein RMSF v. residue (during 20 ns simulation) for (A) hEP4 and (B) rEP4. Smoothed curve over 5 neighboring residues to reduce noise. Red areas indicate alpha helical regions. Green lines indicate ligand contacts. Brown line indicates RMSF for sidechains. Blue line indicates Cα RMSF. Red line indicates B factor, shown only on hEP4 plot. (PNG 132 kb) [file 12860_2019_212_MOESM4_ESM.png]

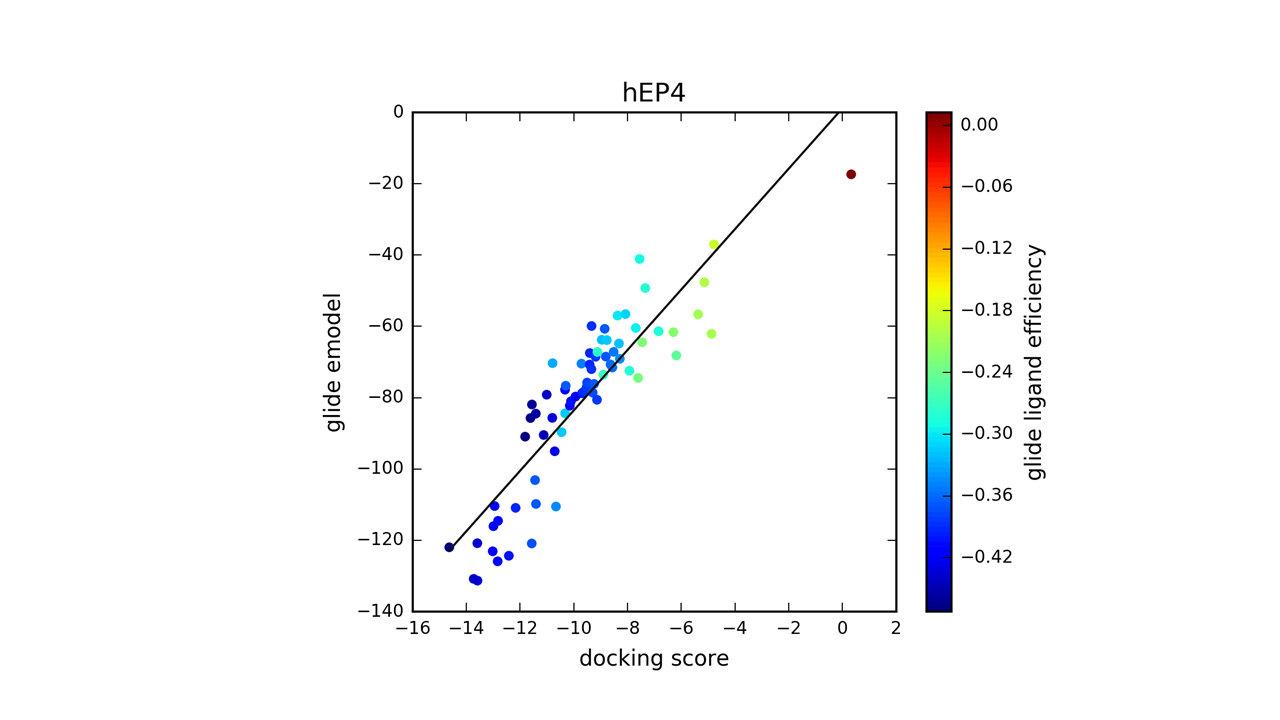

Supplement: Supplementary file 5 — : Figure S5. Representative image of Emodel scores versus Docking score for a set of agonists docked into hEP4. (PNG 77 kb) [file 12860_2019_212_MOESM5_ESM.png]

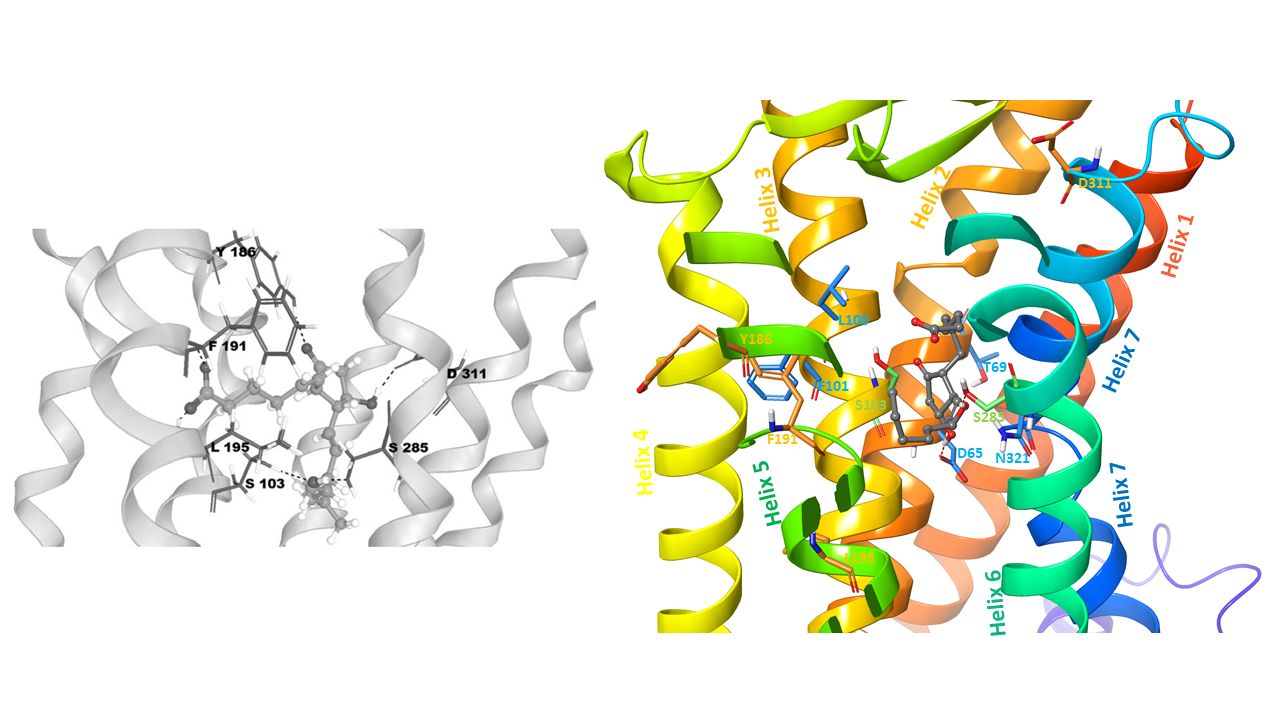

Supplement: Supplementary file 6 — : Figure S6. Side by side comparison of the hEP4 docked PGE2 poses between the Margan model [20] and this study from the same angle. (PNG 525 kb) [file 12860_2019_212_MOESM6_ESM.png]

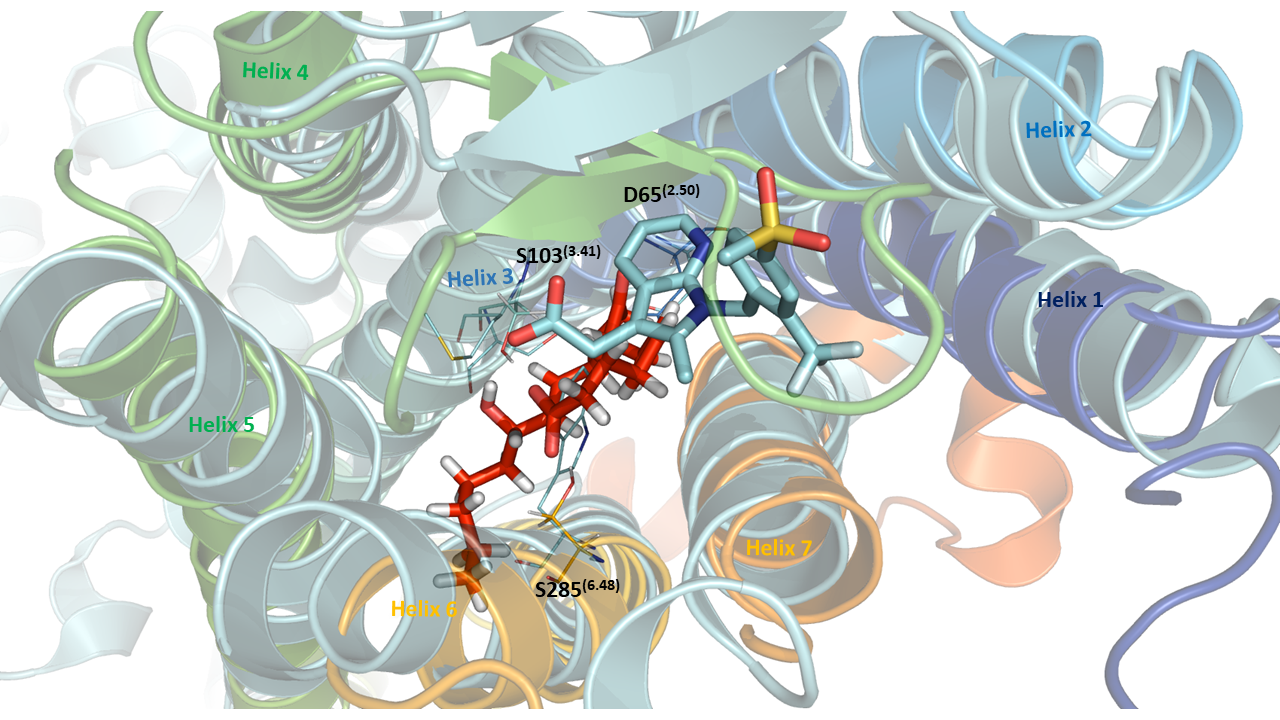

Supplement: Supplementary file 7 — : Figure S7. Alignment of PGE2 docked to hEP4 with Crystal structure of the prostaglandin D2 receptor CRTH2 with fevipeprant. Top down view of receptor with transmembrane helices numbered. hEP4 receptor is display in rainbow coloring. DP2 receptor is shown in teal. Key residues for predicted hEP4 agonist binding site are shown with the corresponding DP2 residues overlaid. C(S/W) xP motif overlays between models with W of the CWxP motif overlaying with S285(6.48). D65(2.50) overlays well between the models. S103(3.41) of the hEP4 structure is closely positioned to either a S118 or M115 residue of DP2 receptor. (PNG 1136 kb) [file 12860_2019_212_MOESM7_ESM.png]

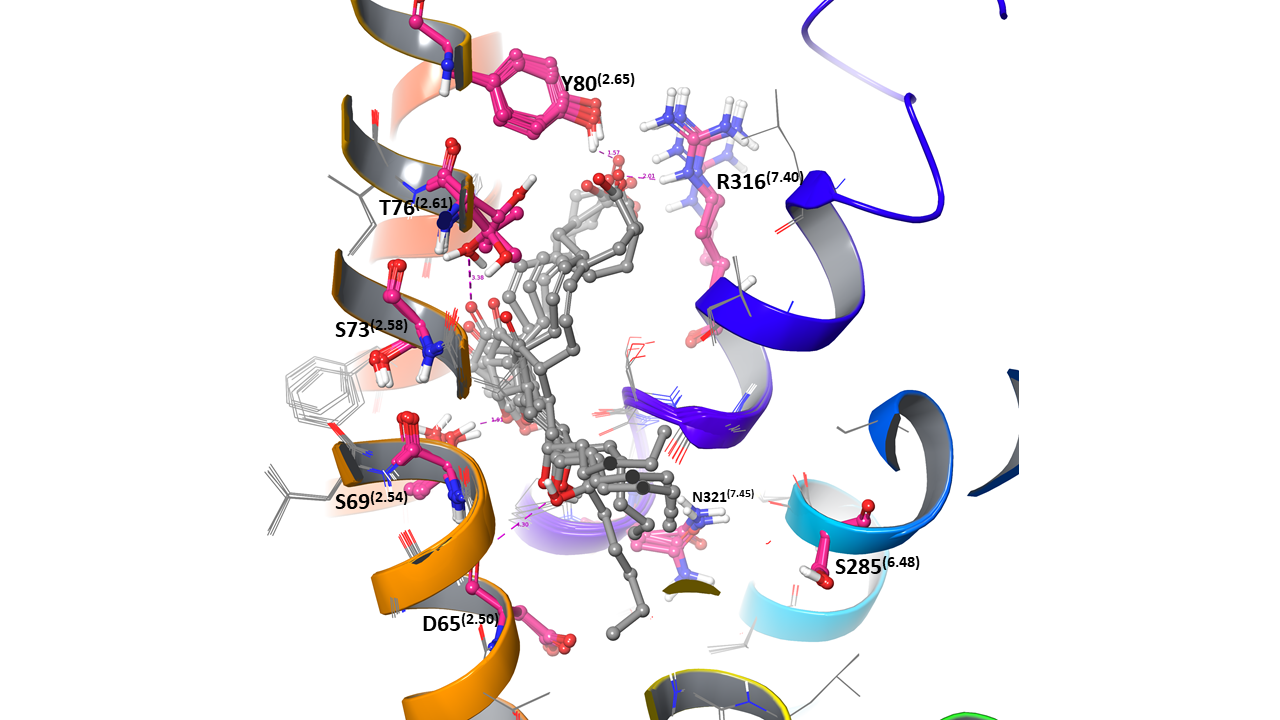

Supplement: Supplementary file 8 — : Figure S8. Homology model of the hEP4 receptor docked with PGE2. All 5 poses output from an induced fit protocol are shown [45, 53–56] . PGE2 is displayed in ball and stick representation with non-polar hydrogens hidden (gray). Dotted lines indicate hydrogen bonding interactions. hEP4-PGE2 interactions include: (1) The alpha chain carboxylate to both Y80(2.65), and R316(7.40) (2) The 11-hydroxyl to the OH group of S69(2.54) (3) The carbonyl oxygen/T76(2.61) and (4) The 15-hydroxyl to D65(2.50) is 4.3 Å and within proximity to the hEP4 backbone. NOTE: This figure is part of the addendum and added to the original manuscript on March 29th, 2019. All other sections of this report were prepared prior to December 5th, 2018 release of EP4 crystal structure. (PNG 451 kb) [file 12860_2019_212_MOESM8_ESM.png]
