# Supplementary material for: Improved homology modeling of the human & rat EP4 prostanoid receptors
Source: BMC Mol Cell Biol. 2019 Aug 27;20:37. doi: 10.1186/s12860-019-0212-5 (PMC6712885; doi:10.1186/s12860-019-0212-5)
Supplement: Supplementary file 9 — : Table S1. RaptorX threading model statistics, before minimized refinement within Maestro. (DOCX 14 kb) [file 12860_2019_212_MOESM9_ESM.docx]

Additional file 9: **Table 1.** RaptorX threading model statistics, before minimized refinement within Maestro.

| **Human RaptorX Template Models** | | | | | |
| --- | --- | --- | --- | --- | --- |
| **Rank** | **P-value** | **Score** | **uGDT/GDT** | **uSeqID/SeqID** | **Template(s)** |
| 1 | 5.3e-07 | 269 | 181/46 | 58/15 | 2ZIY chain a |
| 2 | 4.9e-07 | 270 | 179/46 | 57/15 | 2Z73 chain a |
| 3 | 9.4e-07 | 259 | 166/42 | 51/13 | 4ZWJ chain a |
| 4 | 1.9e-06 | 247 | 167/43 | 54/14 | 4GBR chain a |
| 5 | 4.0e-06 | 234 | 163/42 | 42/11 | 4EIY chain a |
| **Rat RaptorX Template Models** | | | | | |
| **Rank** | **P-value** | **Score** | **uGDT/GDT** | **uSeqID/SeqID** | **Template(s)** |
| 1 | 4.6e-07 | 268 | 180/46 | 57/14 | 2ZIY chain a |
| 2 | 4.4e-07 | 269 | 177/45 | 57/14 | 2Z73 chain a |
| 3 | 8.4e-07 | 258 | 164/42 | 54/14 | 4ZWJ chain a |
| 4 | 1.7e-06 | 246 | 163/41 | 49/12 | 4GBR chain a |
| 5 | 1.2e-06 | 251 | 162/41 | 49/12 | 4MBS chain a |
